# Dataset on pore water composition and grain size properties of bottom sediments and subsea permafrost from the Buor-Khaya Bay (Laptev Sea)

**DOI:** 10.1016/j.dib.2021.107580

**Published:** 2021-11-16

**Authors:** Alexander S. Ulyantsev, Svetlana Yu. Bratskaya, Natalya V. Polyakova, Ivan S. Trukhin, Yulia A. Parotkina

**Affiliations:** a Shirshov Institute of Oceanology, Russian Academy of Sciences, Moscow 117997, Russia; b Institute of Chemistry, Far Eastern Branch of the Russian Academy of Sciences, Vladivostok 690022, Russia

**Keywords:** Arctic, Coastal zone, Bottom sediments, Subsea permafrost, Grain size, Pore water, Major ions

## Abstract

The article presents a dataset on ionic composition of pore water and grain size properties of 105 samples of bottom sediments and subsea permafrost from three sediment cores obtained during polar expeditions in the Buor-Khaya Bay in 2014-2015. Collection sites are located southeast of the Lena Delta near the Bykovsky Peninsula at the Buor-Khaya Bay. In this data article, the concentration of sodium, potassium, calcium, and magnesium cations, chlorides and sulphates in water extracts from sediments, as well as grain size characteristics, are presented. Based on these measurements a difference in salinisation dynamics of thawed strata within the Buor-Khaya Bay is shown.

## Specifications Table

**Table:** 

Subject	Earth and Planetary Sciences
Specific subject area	Subsea permafrost, bottom sediments, pore water
Type of data	Tables Figures
How data were acquired	Samples of bottom sediments and subsea permafrost were collected in the Buor-Khaya Bay during the expeditions to the Arctic in the spring 2014-2015, using drilling apparatus coupled with skidding machine MTCh-4. The ionic composition of pore water was studied by aqueous extraction. The identification of anions was carried out by ion exchange chromatography using a Dionex ICS-5000 analyser. Cations were quantified by atomic absorption spectrometry using a Solaar M spectrophotometer. Grain size parameters were measured via low-angle laser light scattering using a Mastersizer 2000 particle analyser.
Data format	Grain size properties – raw data, concentration of ions in pore water - analysed data (recalculated according to moisture content)
Parameters for data collection	The sampling depths were chosen based on lithology of sediment core. Standard laboratory processing for geochemical and particulate analysis of bottom sediments and subsea permafrost samples.
Description of data collection	Grain size parameters were measured in wet samples. Concentration of cations and anions was measured in water extracts of the dried to constant mass samples.
Data source location	The Arctic Ocean, the Laptev Sea, Buor-Khaya Bay. GPS coordinates of the drilling wells and the map are provided in Table 1 and Figure 1 respectively.
Data accessibility	Data identification number: 10.17632/y6y6cg94dw.3 [1] The data is available on Mendeley Repository online: http://dx.doi.org/10.17632/y6y6cg94dw.3
Related research article	A.S. Ulyantsev, N.V. Polyakova, E.A. Romankevich, I.P. Semiletov, V.I. Sergienko, Ionic composition of pore water in shallow shelf deposits of the Laptev Sea, Dokl. Earth Sci. 467 (2016). https://doi.org/10.1134/S1028334x16030211.

## Value of the Data

• Concentration of major cations and anions in pore water coupled with grain size characteristics represent an important data in postglacial sedimentation study.
• Published data will be useful for characterization the salinization rates of the sedimentary strata in the Arctic shelf. It will be useful for geologists, geochemists, geocryologists, and researchers who work with Arctic objects.
• Under the conditions of climate change in the Arctic, a comparative analysis of the grain size and pore water in bottom sediments and permafrost rocks is of great importance for understanding dynamics and mechanisms of subsea permafrost thawing.

## 1 Data Description

**Fig. 1:**
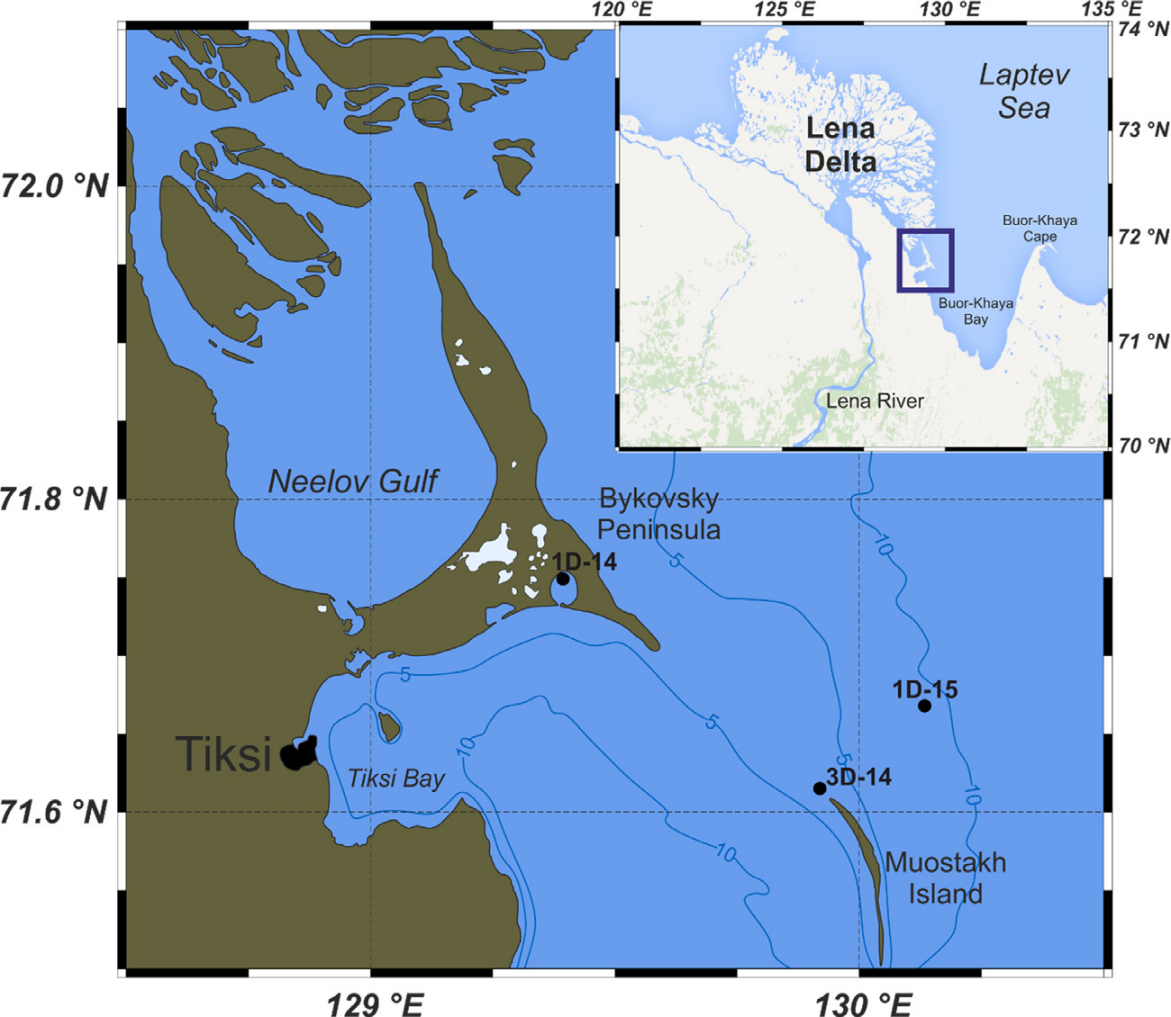
Location of the collection sites within Buor-Khaya Bay in 2014 (1D-14 and 3D-14 boreholes) and 2015 (1D-15 borehole).

**Fig. 2:**
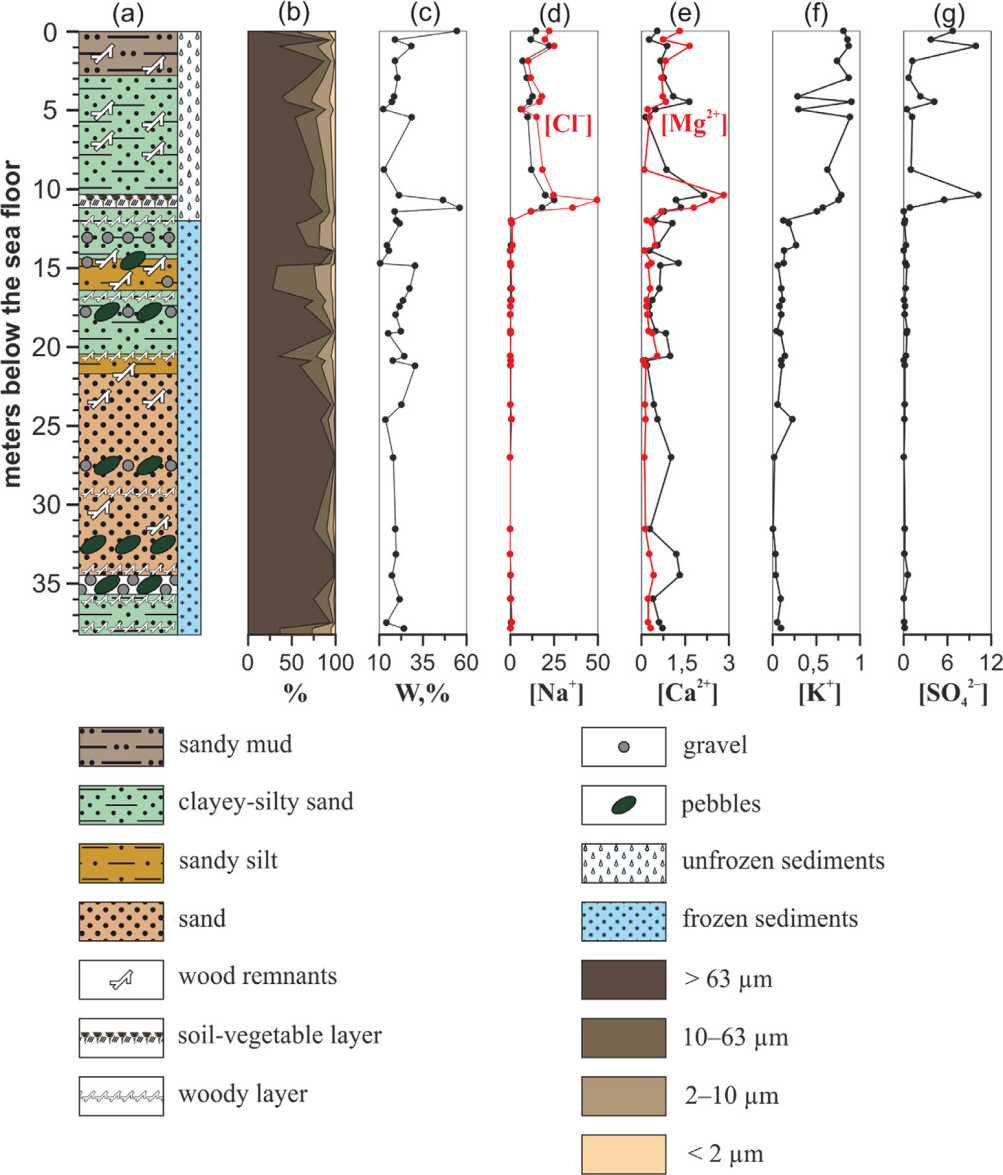
Geological characteristics and calculated concentration of ions in aqueous extracts of samples from 1D-14 sediment core. (a) – lithology, (b) – grain size, (c) – moisture content, (d) – concentration of sodium cations (black) and chlorides (red), (e) – concentration of calcium (black) and magnesium (red) cations, (f) – concentration of potassium cations, (g) – concentration of sulphates. Concentration of ions is expressed in g/L.

**Fig. 3:**
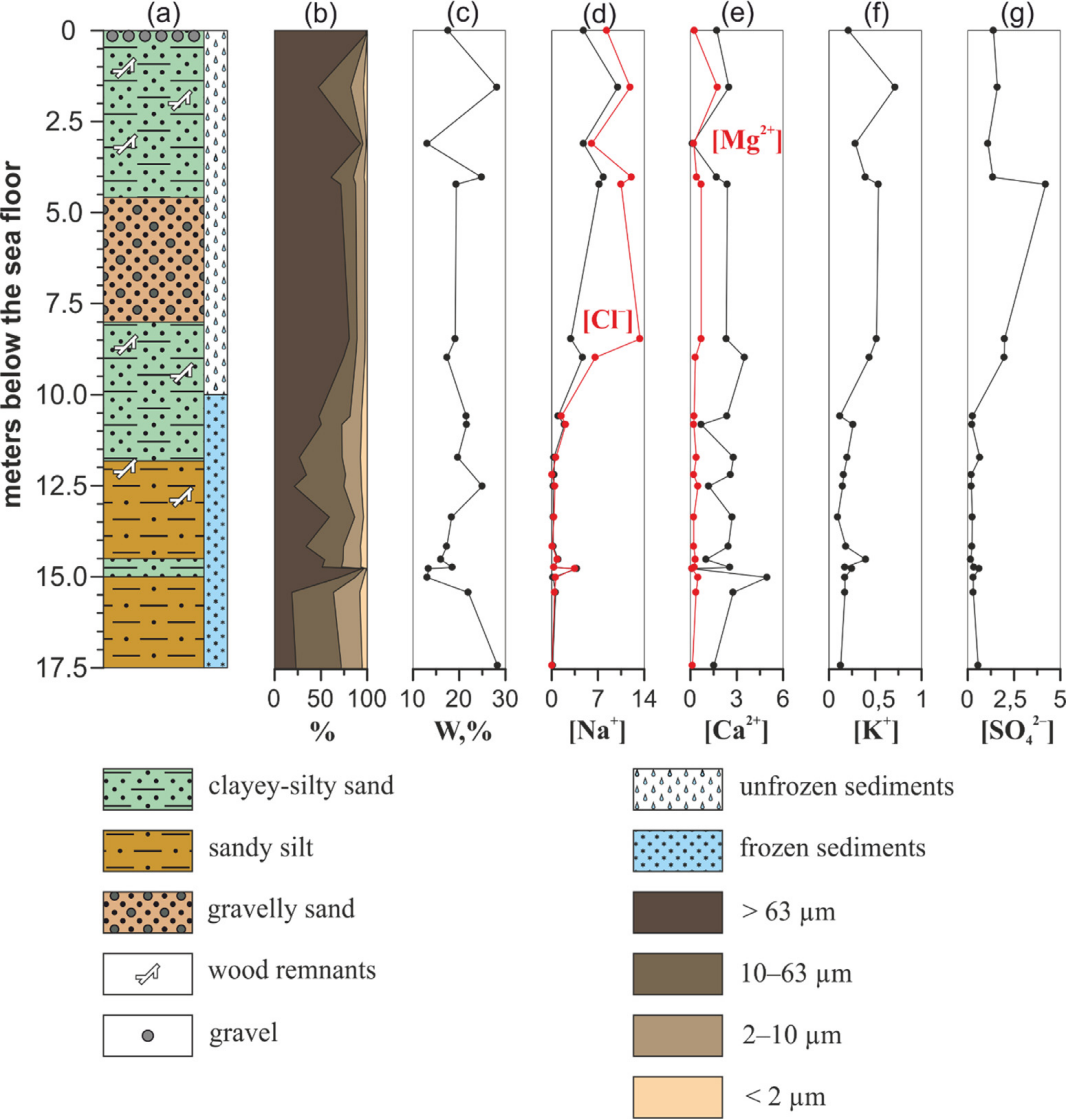
Geological characteristics and calculated concentration of ions in aqueous extracts of samples from 3D-14 sediment core. (a) – lithology, (b) – grain size, (c) – moisture content, (d) – concentration of sodium cations (black) and chlorides (red), (e) – concentration of calcium (black) and magnesium (red) cations, (f) – concentration of potassium cations, (g) – concentration of sulphates. Concentration of ions is expressed in g/L.

**Fig. 4:**
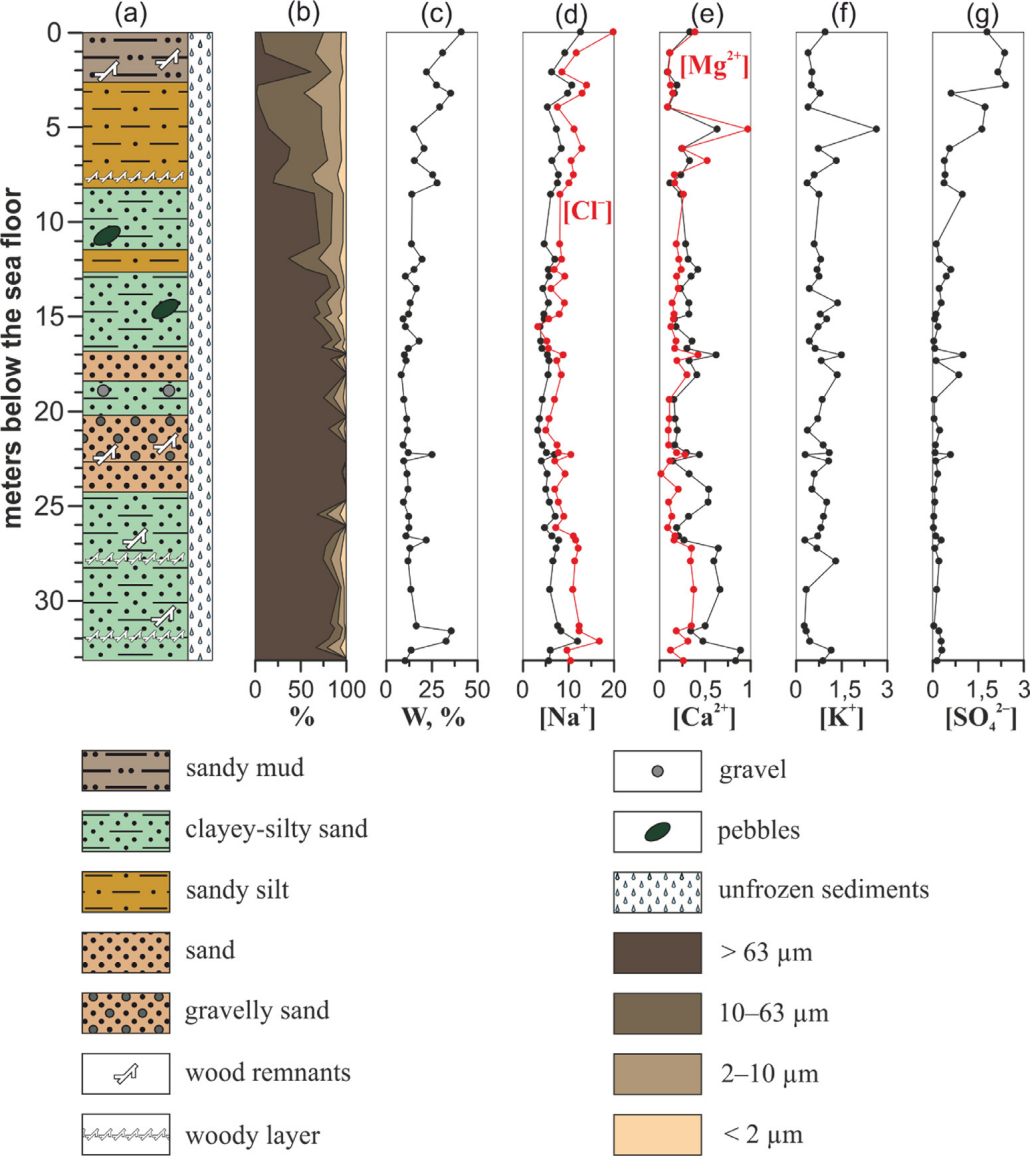
Geological characteristics and calculated concentration of ions in aqueous extracts of samples from 3D-14 sediment core. (a) – lithology, (b) – grain size, (c) – moisture content, (d) – concentration of sodium cations (black) and chlorides (red), (e) – concentration of calcium (black) and magnesium (red) cations, (f) – concentration of potassium cations, (g) – concentration of sulphates. Concentration of ions is expressed in g/L.

Three drilling cores were obtained during polar expeditions in 2014 (1–14 April) and 2015 (28 March–14 April) which were organised by the Pacific Oceanological Institute FEB RAS (Vladivostok), the National Research Tomsk Polytechnic University (Tomsk), and the Melnikov Permafrost Institute SB RAS (Yakutsk), in collaboration with Lomonosov Moscow State University (Moscow) and Shirshov Institute of Oceanology RAS (Moscow) [2]. Sediment cores were collected in the Buor-Khaya Bay using drilling apparatus PBU-2 coupled with skidding machine MTCh-4 [3]. Collection sites are shown in Fig. 1. Table 1 contains coordinates of sampling and brief drilling core data. Figs. 2–4 show lithology, grain size and calculated concentration of major ions in the pore water of the studied sediment cores. The dataset from Mendeley repository contain Table 1, Figs. 1–4, Excel file with primary data and folder with images of salinised moss from 1D-14 sediment core.

**Table 1:** GPS coordinates and field characteristics of the drilling wells.

Sediment core	Latitude, ° N	Longitude, ° E	Well depth, m	Ice thikness, m	Water thickness, m	Permafrost roof, m
1D-14	71.755	129.397	38.2	1.8	1.3	12
3D-14	71.619	129.916	17.5	1.7	1.0	10
1D-15	71.672	130.137	33.2	1.6	7.9	not passed

## 2 Experimental Design, Materials and Methods

The sampling depths were chosen based on lithology of each sediment core. Unfrozen sediments were sampled with a steel spatula. Sampling of consolidated permafrost deposits from the drilling cores was carried out with the electric screwdriver and rathole bits. All samples were put into zip-lock polypropylene bags and kept frozen at –20°C for analytical procedures. The number of samples was as follows: 38 samples of core 1D-14, 19 samples of core 3D-14, and 48 samples of core 1D-15.

The ionic composition of pore water was studied by aqueous extraction. Wet subsamples of deposits were dried with a MOC-120H moisture analyser (Shimadzu, Japan) at 105°C for a constant mass. The dried samples (approx. 20 g) were placed into glass flasks and 150 ml Milli-Q water was added. Flasks with suspended samples were shaken with a Vortex Genius 3 shaker (IKA, Germany) for 15 min. Then, the water that was extracted was centrifuged using a 5702R centrifuge (Eppendorf, Germany) at 5500 rpm for 10 min to precipitate solids. Quantification of cations (sodium, potassium, calcium, magnesium) and anions (chlorides and sulfates) was performed in supernatants.

The identification of anions was carried out by ion exchange chromatography with conductometric detection using a Dionex ICS-5000 analyser (Termo Scientific, USA) with pre-column and column PAX-100. A mix of carbonate buffer solution (3/4 mM of sodium carbonate/hydrocarbonate). Acetonitrile (5%) was used as an eluent. Cations were quantified by atomic absorption spectrometry using a Solaar M spectrophotometer (Termo Scientific, USA). Water extracts were diluted in 100 times for sodium and potassium quantification, in 20 times for calcium, and in 10 times for magnesium resulting analytical in an range from 1 to 30 mg/L. Wavelengths were 766.5 nm for potassium, 589.0 nm for sodium, 422.7 nm for calcium, and 285.2 for magnesium, flame – mix of acetylene and air. Spectral and chemical noise was eliminated by adding the buffer solutions: 0.1% of Cs for K and 0.2% of La for Ca and Mg. Precision for cations was at ≤ 10%. The results were recalculated considering a dilution rate and moisture content, and expressed as g/L.

Grain size parameters of deposits were measured via low-angle laser light scattering in wet samples (approx. 20 g) using a Mastersizer 2000 particle analyser (Malvern Instruments, UK) according to ISO 13320:2009. Analytical conditions were 2000 rpm pump speed, 25 W ultrasound sonification power (40 KHz), 30 s exposition time for one measurement, and 2500 Hz scanning frequency. Milli-Q water was used as a dispersant and a blank. All laser diffraction analyses were done in triplicate and the results were averaged. The relative mass contributions (in%) of size fractions were expressed as follows: >63 µm, 10–63 µm, 2–10 µm and <2 µm.

## CRediT authorship contribution statement

**Alexander S. Ulyantsev:** Writing – original draft, Visualization, Project administration, Conceptualization, Funding acquisition. **Svetlana Yu. Bratskaya:** Investigation. **Natalya V. Polyakova:** Formal analysis, Validation. **Ivan S. Trukhin:** Investigation. **Yulia A. Parotkina:** Investigation.

## Declaration of Competing Interest

The authors declare that they have no known competing financial interests or personal relationships that could have appeared to influence the work reported in this paper.
